# Synergistic adsorption-photocatalytic degradation effect and norfloxacin mechanism of ZnO/ZnS@BC under UV-light irradiation

**DOI:** 10.1038/s41598-020-68517-x

**Published:** 2020-07-17

**Authors:** Wen Liu, Tianpei He, Yonghong Wang, Ge Ning, Zhenggang Xu, Xiaoyong Chen, Xinjiang Hu, Yaohui Wu, Yunlin Zhao

**Affiliations:** 1 College of Life Science and Technology, Central South University of Forestry and Technology, Changsha, 410004 China; 2 International Education Institute, Hunan University of Chinese Medicine, Changsha, 410208 China; 3 College of Arts and Sciences, Governors State University, University Park, IL 60484 USA

**Keywords:** Pollution remediation, Photocatalysis

## Abstract

Norfloxacin (NOF) is an environmentally harmful and ubiquitous aquatic pollutant with extensive production and application. In this study, a novel composition named carbon-based composite photocatalytic material of zinc oxide and zinc sulphide (ZnO/ZnS@BC) was successfully obtained by the impregnation-roasting method to remove NOF under UV-light. Scanning electron microscopy, X-ray photoelectron spectroscopy, transmission electron microscopy and energy dispersive spectrometer characterised the composition. ZnO/ZnS was successfully decorated on the surface of biochar (BC). The pH, the ZnSO_4_/PS ratio, and ions and quenchers, were investigated. High removal efficiency was obtained with a pH of 7 and a ZnSO_4_/PS ratio of 1:1, and the removal ratio of NOF reached 95% within three hours; the adsorption and degradation ratios reached 46% and 49%, respectively. Fe^2+^ promoted the degradation of NOF, whereas other ions inhibited it, with NO_3_^−^ showing the strongest inhibitory effect. Three reactive species (tert-butanol, quinone, and ammonium oxala) were identified in the catalytic system. The decreasing order of the contribution of each reactive species was: O_2_^−^ > ·OH^−^ > h^+^. Additionally, a recycling experiment demonstrated the stability of the catalyst; the catalytic degradation ratio of NOF reached 78% after five successive runs. Therefore, ZnO/ZnS@BC possessed strong adsorption capacity and high ultraviolet photocatalysis ability.

## Introduction

As secondary metabolites produced by animals, plants, or microorganisms (bacteria, fungi, and actinomycetes), antibiotics are resistant to pathogens and have been increasingly used for the prevention and treatment of diseases in humans and animals. Serious antibiotic contamination can damage the microflora balance in the environment and in humans^1^. Norfloxacin (NOF) is widely used because of its broad-spectrum antibacterial and low-cost characteristics and is frequently detected in water environments. NOF blocks the action of DNA gyrase in pathogenic bacteria and destroys the entire ecosystem balance^2^. Therefore, it is necessary and urgent to find an effective and rapid method for the remove of antibiotics from the aquatic environment^3, 4^.

Traditional methods such as adsorption^5, 6^, oxidation^7, 8^, and photocatalysis^9, 10^ have been used to remove NOF from water and produce certain effects. The light-sensitive character of NOF makes photocatalytic degradation a common method. Zinc oxide (ZnO), an n-type semiconductor, is one of the most interesting photocatalysts because of its relatively large excitation binding energy (60 eV) and wide band gap (3.37 eV). ZnO is primarily activated by ultraviolet light with a wavelength below 385 nm^11^. Hu successfully synthesised multilayer ZnO nanoflowers, which degrade NOF well under ultrasound (US) irradiation^12^. However, ZnO catalysts have some shortcomings, such as easy electron–hole pair reset and catalyst aggregation. Multiple efforts have been made to enhance the photocatalytic properties of ZnO. Methods reported in literature to improve the activity primarily include (I) combining ZnO with other chemical compounds to compose new mixed photocatalysts with different band gap sizes^13^ and (II) loading ZnO onto carbon materials that increase ZnO dispersion and electron conduction^14^. On the one hand, two individual photocatalysts could obtain two-step excitation by an electron transfer mediator^15^. Sulphides are good photocatalyst candidates, and many wide band gap semiconductors (ZnS^16^, CdS^17^, and ZnSe^18^) were utilised to synthesize ZnO-based photocatalysts. ZnS attracted more attention because it is nontoxic and chemically stable and has wide band gap (3.72 eV) characters^19^. ZnO/ZnS has been demonstrated to have improved physical and chemical properties^20^. Recent research shows that changing the structure and properties of materials improves the catalytic performance^21–23^. Bo reported a good photocatalytic activity chip perpendicular to ZnS-coated ZnO nanorod arrays (ZnO@ZnS NAs) that effectively degrade tetracycline hydrochloride in water; the degradation rate was 62% and increased by 14% compared to that obtained with ZnO^24^. Ag/ZnS composite material has good photodegradability for methylene blue (the degradation rate was 90%) and enables a 1.3-fold enhancement in photocatalytic performance compared to ZnS^25^.

On the other hand, because of its large special surface and good mechanical and unique electronic properties, carbon possesses wide potential applications and can be used as a carrier for the catalyst to conduct electrons and disperse catalyst particles. This is a crucial step in the large-scale application of the photocatalytic technique^26^. Ag and ZnO nanoparticles can be uniformly dispersed on the surface of the carbonaceous layers to form Ag/ZnO/C composites, which can remove tetracycline hydrochloride. Additionally, Ag/ZnO/C possesses a higher adsorption capacity and greater photocatalytic activity than ZnO/C and pure ZnO^27^.

**Figure 1:**
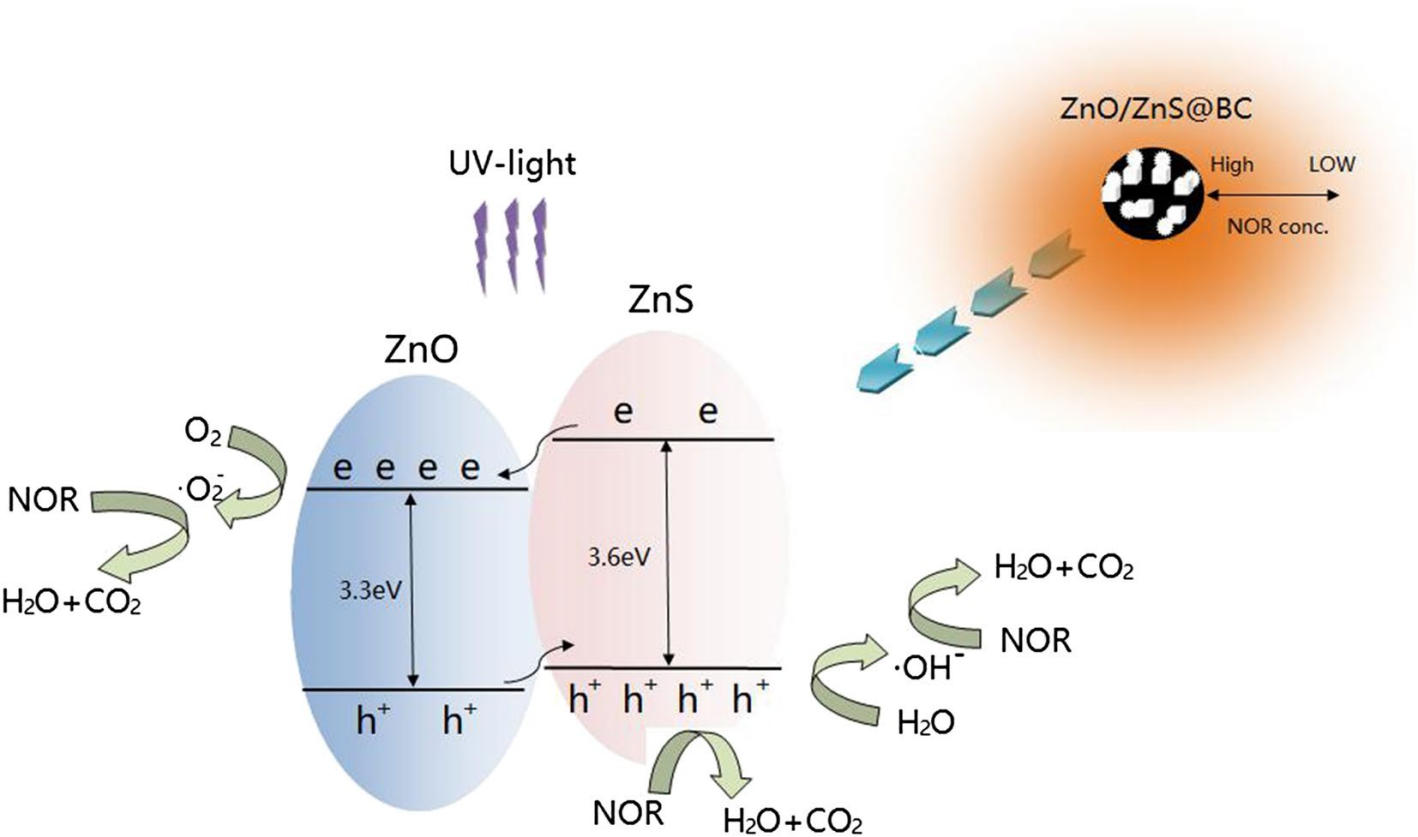
Schematic diagram of the working principle of ZnO/ZnS@BC.

Biochar (BC) is easily prepared, cost-effective, and eco-friendly and exhibits excellent biological, electrical, and optical properties^28^. It possesses considerable potential application in the photocatalyst field. In our research, the composite catalyst ZnO/ZnS@BC was synthesised by the impregnation-roasting method. The primary goals were to: (1) characterise the ZnO/ZnS@BC structure and (2) investigate the adsorption-photocatalysis and antibiotics degradation mechanism of ZnO/ZnS@BC for NOF degradation under UV-light. The ZnO/ZnS@BC might increase norfloxacin adsorption and enhance norfloxacin degradation (Fig. 1).

## Materials and methods

### Chemicals

Poplar sawdust (PS) was supplied by Shouite Hongfa (Beijing) Biomass Energy Technology Co. Ltd. China. NOF (99.9%) was purchased from Aladdin–Holdings Group, China. Hydrochloric acid (HCl) was purchased from Guangdong Huada Energy Co. Ltd. China. Sodium nitrate (NaNO_3_) was purchased from Xinchuan Chemical Co. Ltd. China. Sodium hydroxide (NaOH), sodium sulphate (Na_2_SO_4_), ferrous sulphate (FeSO_4_), zinc sulphate (ZnSO_4_), sodium carbonate (Na_2_CO_3_), cupric sulphate (CuSO_4_), sodium chloride (NaCl), sodium citrate (Na_3_C_6_H_5_O_7_), benzoquinone (C_6_H_4_O_2_), ammonium oxalate (C_4_H_10_O), and tert-butanol (NH_4_)_2_C_2_O_4_) were purchased from Behringer Technology Co. Ltd. Germany. All reagents were applied without further purification and were of analytical grade.

### Preparation and characterisation of the catalysts

The photocatalysts were prepared by the one-step synthesis method. The Poplar sawdust was pulverised, placed in a 100 mesh sieve, and intruded into a zinc sulphate (ZnSO_4_) solution at 60 °C water bath for 5 h. The sawdust was then placed in an oven for drying at 80 °C for 12 h and finally carbonised in a muffle furnace at 600 °C for 2 h to produce ZnO/ZnS@BC.

Scanning electron microscopy (SEM, SU8010, Hitachi) and transmission electron microscopy (TEM, HF5000, Hitachi) were used to observe the microscopic morphology of the catalyst loaded on carbon. X-ray photoelectron spectroscopy (XPS, BIR-KV201 and Japan) and energy dispersive spectrometer (EDS, Octane SDD, EDAX) were used to investigate the elemental and chemical valence states of carbon-based composite photocatalysts.

### Degradation and reuse experiments

A certain amount of catalysts and 50 mL of NOF solution were added into the 250 mL beaker, which was placed on a 160-rpm shaker for the photocatalytic degradation experiments, which were performed in a confined space with UV lamps. HCl (0.1 M) and NaOH (0.1 M) were used to adjust the pH. All the parameters except the investigated parameter were fixed as follows: pH 7, NOF 0.025 g L^−1^, ZnO/ZnS@BC 0.5 g L^−1^, and temperature of 25 °C. The experiments were carried out at least thrice, and the results were averaged. 10 mg L^−1^ benzoquinone, ammonium oxalate and tert-butanol were added as hydroxyl radical scavengers when needed to explore the catalytic mechanism.

### Analytic methods

The concentration of NOF was detected by high-performance liquid chromatography (HPLC) (Agilent 1200 Series, Agilent, USA). The octadecylsilane-bonded silica gel was used as filler. The mobile phase for NOF was a mixture of 0.025 M phosphoric acid solution (adjust the pH to 3.0 ± 0.1 with triethylamine) and acetonitrile (87:13 (v/v), and the detection wavelength was 255 nm. The flow rate was 1 mL min^−1^, and the column temperature was 30 °C.

## Results and discussion

### Structural properties

**Figure 2:**
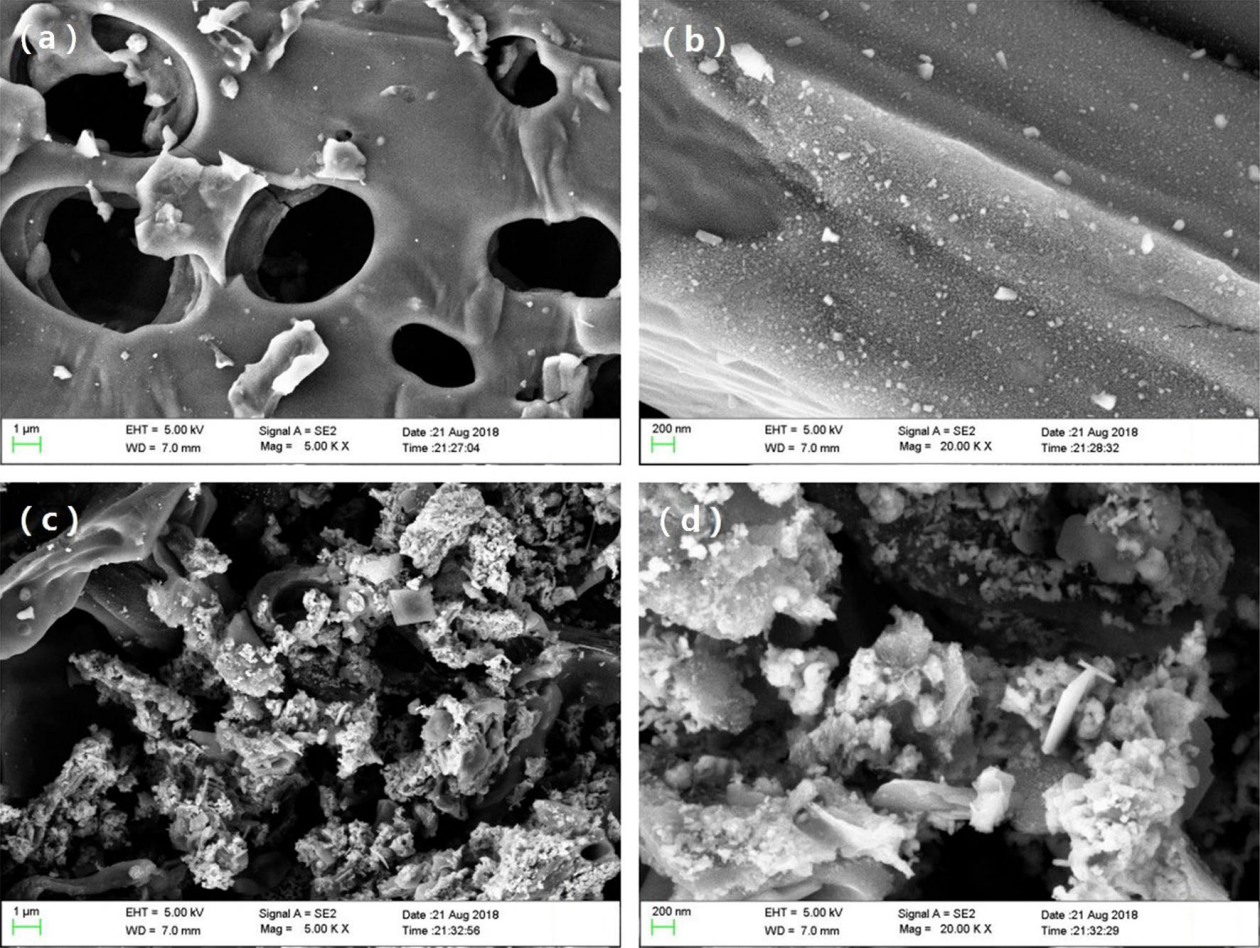
SEM images of the samples: (**a–b**) BC; (**c–d**) ZnO/ZnS@BC.

The microscopic morphology of the BC and ZnO/ZnS@BC was analysed by SEM (Fig. 2). Figure 2a,b shows the abundant circular pore structure of the BC without loaded particulate matter on the surface. Compared with BC, the surface of the ZnO/ZnS@BC (Fig. 2c,d) was loaded with abundant white particles that were irregular circles and tetragonal crystals with diameters ranging from tens to hundreds of nanometres. The particles were successfully supported on the surface of the BC.

**Figure 3:**
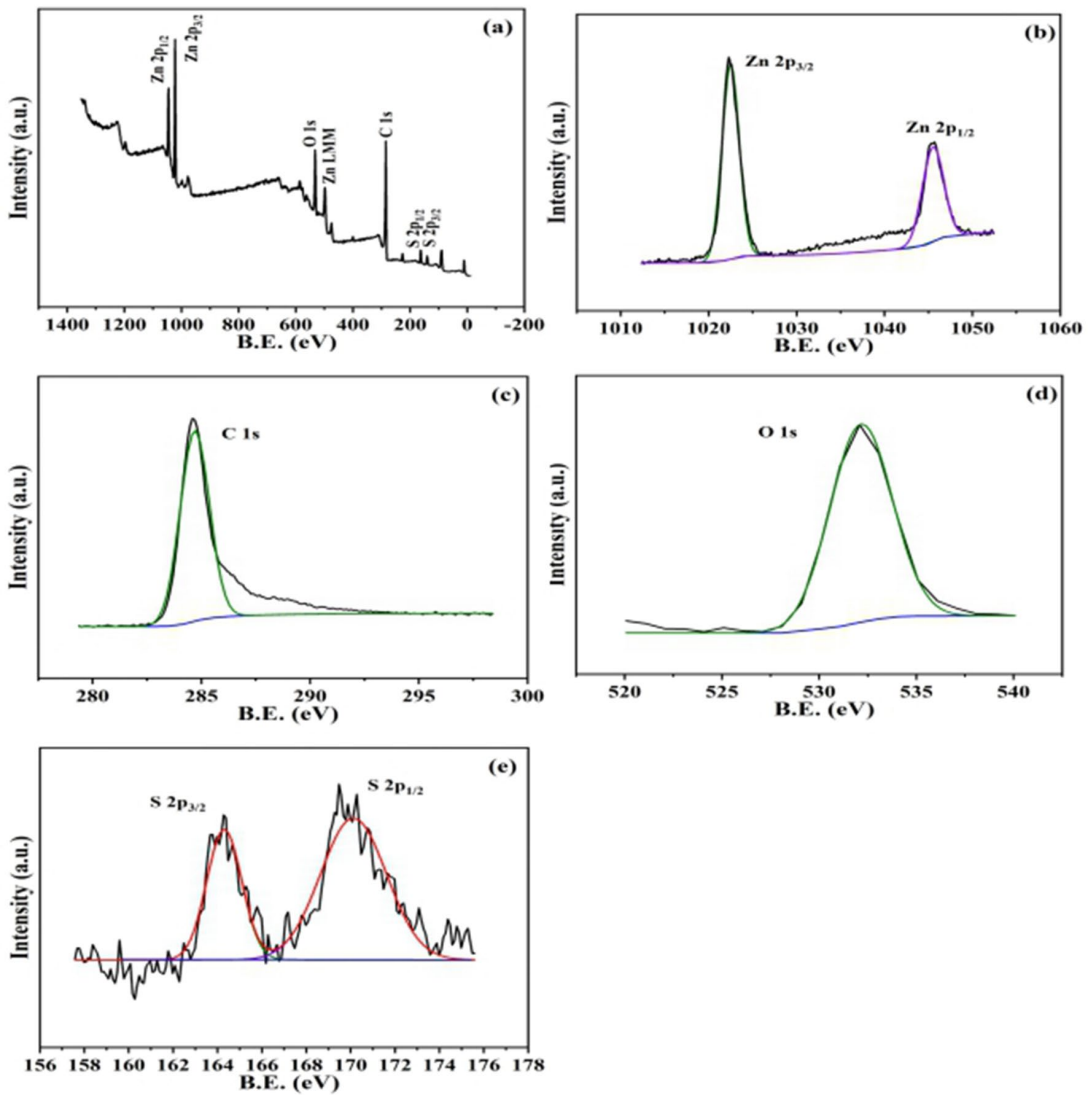
XPS spectrum of ZnO/ZnS@BC.

XPS analysis was used to characterise Zn 2p, S 2p, O 1s, and C 1s to investigate the element and chemical valence states of carbon-based composite photocatalysts. Figure 3 shows the full spectrum of the carbon-based composite photocatalyst, which contained the characteristic peaks of Zn 2p, S 2p, C 1s, and O 1s, indicating that the carbon-based composite photocatalyst possessed Zn, S, O, and C elements. The peaks near 1,021.75 and 162.7 eV were the binding energies of Zn2p and S2p1/2, respectively, indicating the presence of Zn–S bonds in the catalyst. This was consistent with the value of the Zn–S bond in other reports^29, 30^. There was an O 1s peak near 532.0 eV. According to other studies, the surface O 1s peak was at 530.0 eV in pure ZnO^31, 32^. Compared to other literature, ZnO/ZnS exhibited a larger O 1s peak area near 532.0 eV and lattice oxygen intensity, which could be attributed to the aggregation of ZnS and ZnO^26^. Therefore, the heterojunction of ZnS and ZnO likely existed in the catalyst. Combined with SEM results analysis, the chemical composition of the white particles should be ZnO and ZnS. The heterojunction of ZnS and ZnO was loaded onto the BC.

**Figure 4:**
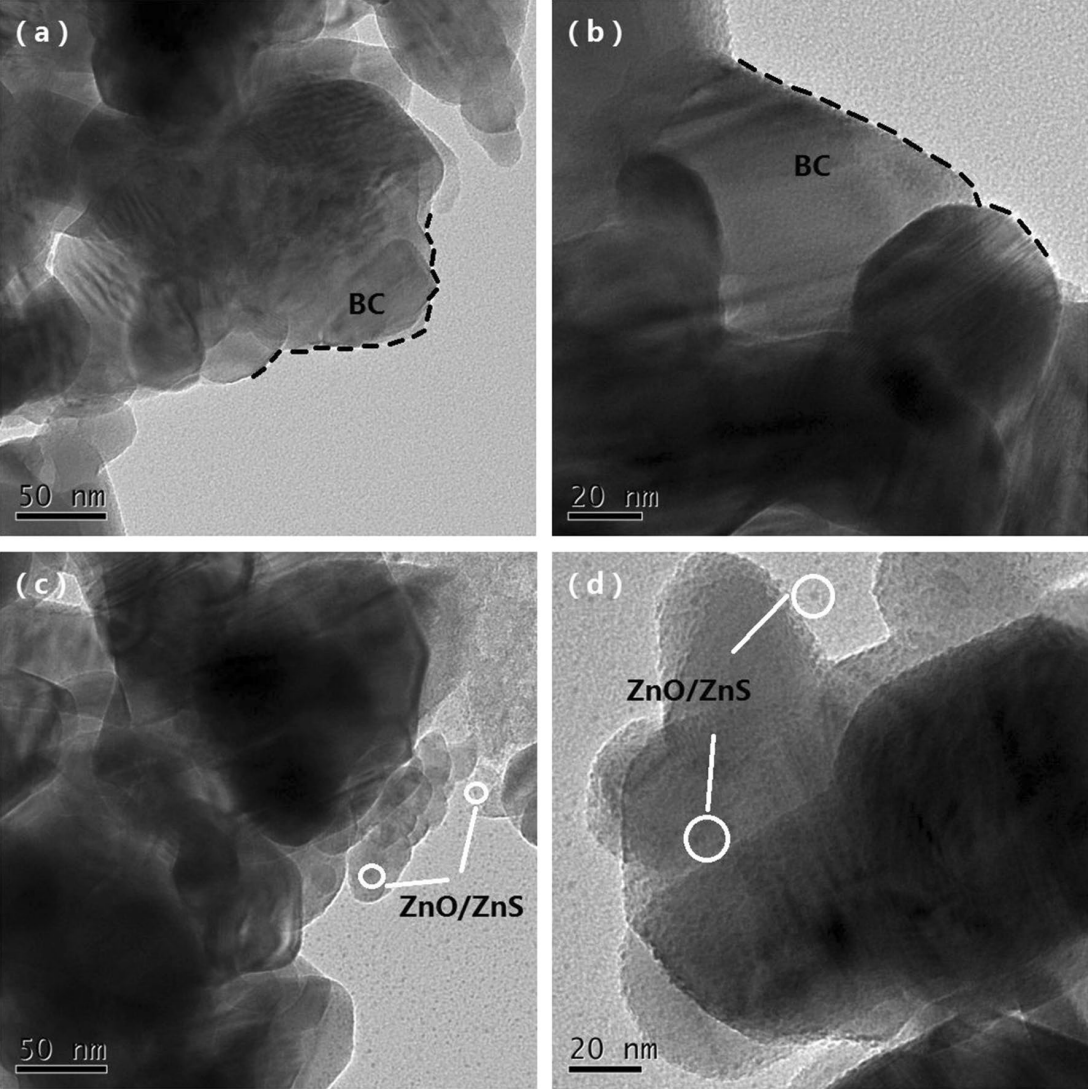
TEM images of the samples: (**a**,**b**) BC; (**c**,**d**) ZnO/ZnS@BC.

Figure 4 shows the TEM image of the BC and ZnO/ZnS@BC and reveals detailed information about the microstructure of photocatalyst. There are no dark spots on the surface of the BC Fig. 4a,b, but darker spots appear on the surface of the BC in Fig. 4c,d. According to the XPS results, the darker spots might be ZnO and ZnS. Additionally, the grain size of the ZnO/ZnS composite was approximately 2 nm, and the ZnO/ZnS had an irregular shape.

**Figure 5:**
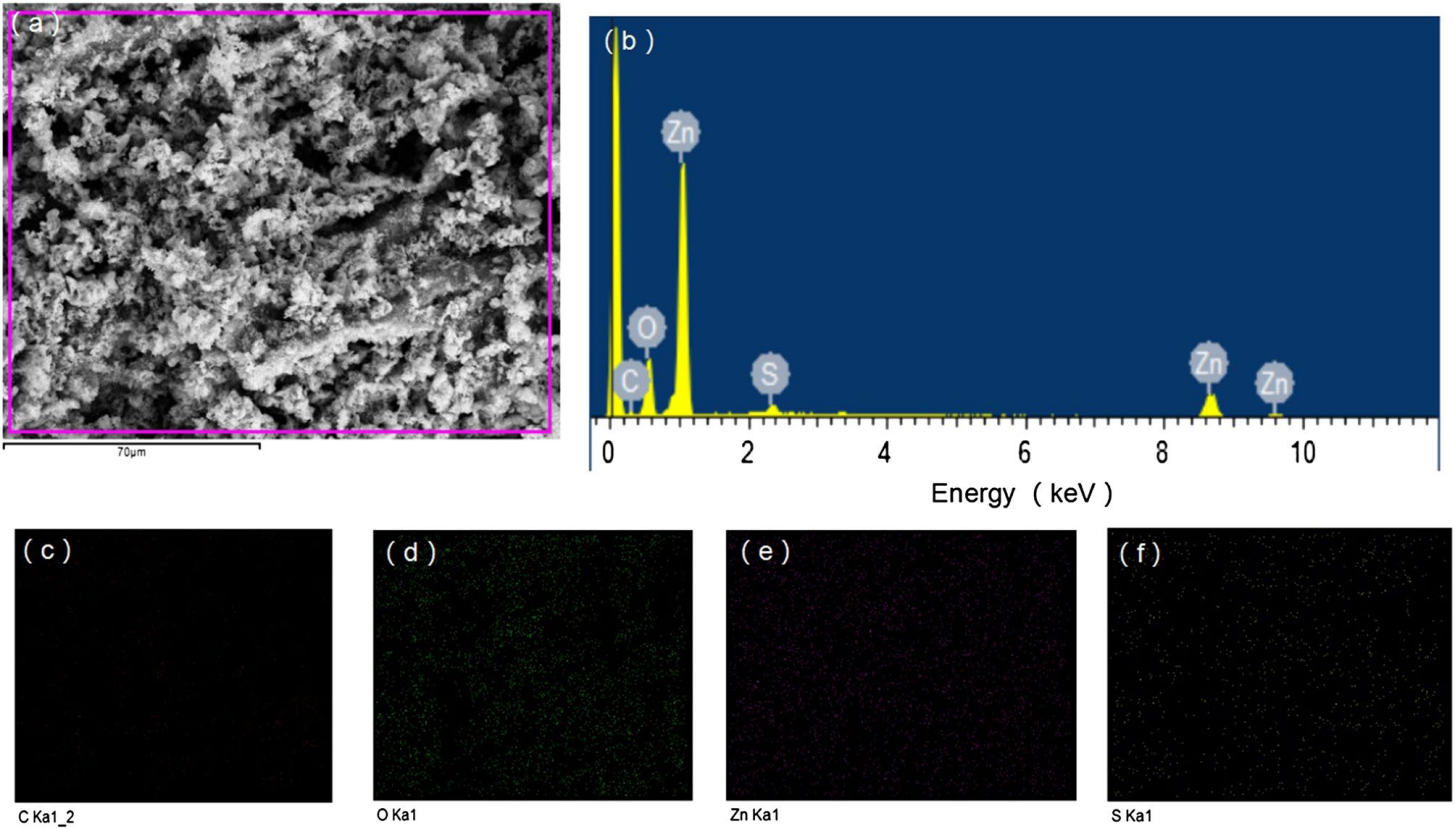
EDS images of the samples: (**a**) SEM image; (**b**) EDS spectrum; (**c**–**f**) overlay elemental mapping images of ZnO/ZnS@BC: C (red), O (green), Zn (purple) and S (yellow).

EDS was an effective method to analyze the chemical composition of nanocomposites^33^. The microstructures and element distribution of ZnO/ZnS@BC photocatalyst were further studied by SEM and EDS mapping (Fig. 5a–f), respectively. The Fig. 5b indicated that the sample contains C, O, Zn and S elements, and Fig. 5c–f confirmed that C (red), O (green), Zn (purple) and S (yellow) elements were uniformly distributed in the heterojunction. Therefore, ZnO/ZnS and BC were intimately combined.

### Degradation of NOF under different conditions

**Figure 6:**
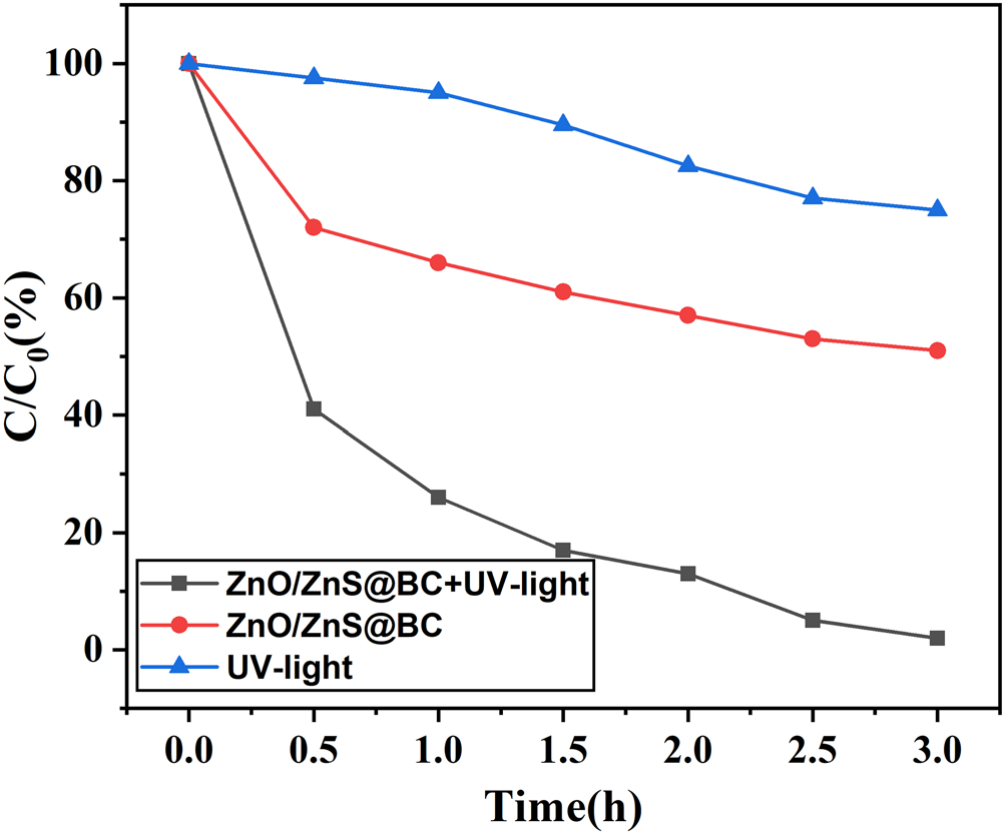
Degradation of NOF under different conditions ([NOF] = 0.025 g L^−1^; [ZnO/ZnS@BC] = 0.5 g L^−1^; pH = 7).

Feasibility experiments were performed to investigate the effectiveness of the ZnO/ZnS@BC in degrading NOF under UV-light. Figure 6 shows the removal rate of NOF under various experimental conditions, where C is the concentration of NOF remaining in the solution after irradiation time t, and Co is the initial concentration of NOF. The ZnO/ZnS@BC and UV-light system removed 95% of the NOF, whereas the dark ZnO/ZnS@BC system removed 45% of the NOF, suggesting that the NOF was removed by adsorption. Under UV-light without the ZnO/ZnS@BC system, 20% of the NOF was degraded after 3 h, indicating that the NOF was not degraded by UV-light. The results showed that the NOF removal rate was highest in the ZnO/ZnS@BC and UV-light system. These results indicated that NOF removal is a synergistic process. BC possessed abundant adsorption sites and quickly attracted NOF to the surface. ZnO/ZnS had strong catalytic capability and quickly degraded the NOF. The presence of BC effectively prevented the aggregation of ZnO/ZnS.

### Kinetic study of NOF degradation by a ZnO/ZnS@BC and UV-light system

Kinetics help understand the mechanisms of pollutant degradation. Recent research reports that the kinetics of the photocatalytic degradation of aqueous pollutants follows the pseudo-first-order kinetic model^34^, and the integration of the pseudo-first-order kinetic model is the following equation:

$${\ln}\frac{{{\text{C}}_{{0}} }}{{{\text{C}}_{{\text{t}}} }} = {\text{k}}_{0} {\text{t}}$$

where C_0_ is the initial concentration of NOF, C_t_ is the concentration of NOF at time t, k_0_ is the pseudo-first-order reaction rate constant (min^−1^), and t is the reaction time (min). The reaction rate constant (k) is calculated from the slope of a plot of ln (C_0_/C_t_) versus (t).

The influence of various parameters such as initial pH, the ratio of ZnSO4:PS (m:m), and ZnO/ZnS@BC concentration on the kinetics of NOF degradation were investigated. Table 1 presents the values of the kinetic rate constants (k_0_) related to the various parameters and their regression coefficients R^2^. The photocatalytic degradation approximately followed the pseudo-first order kinetics (Table 1). The NOF degradation rate constant was highest (0.021 min^−1^) With a pH of 7, ZnSO_4_:PS (m:m) ratio of 1:1, and 25 mg ZnO/ZnS@BC.

**Table 1:** Influence of various parameters on the kinetic of NOF degradation.

Parameter	Value	Equation	*K*_0_ (min^−1^)	*R*^2^
Initial pH	3	Y = 0.0087x + 0.2482	8.7 × 10^–3^	0.9392
	5	Y = 0.0083x + 0.2964	8.3 × 10^–3^	0.9058
	7	Y = 0.0212x + 0.0686	2.12 × 10^–2^	0.9917
	9	Y = 0.0085x + 0.2420	8.5 × 10^–3^	0.9023
	11	Y = 0.0091x + 0.0598	9.1 × 10^–3^	0.9859
ZnSO4:PS (m:m)	1:0.5	Y = 0.0086x + 0.2921	8.6 × 10^–3^	0.8998
	1:1	Y = 0.0171x + 0.0979	1.71 × 10^–2^	0.9815
	1:2	Y = 0.0072x + 0.0584	7.2 × 10^–3^	0.9842
	1:3	Y = 0.0042x + 0.0647	4.2 × 10^–3^	0.9473
	1:4	Y = 0.0030x + 0.0263	3.0 × 10^–3^	0.9344
ZnO/ZnS@BC concentration	5	Y = 0.0034x + 0.1883	3.4 × 10^–3^	0.9015
	10	Y = 0.0045x + 0.2314	4.5 × 10^–3^	0.9325
	15	Y = 0.0050x + 0.2409	5.0 × 10^–3^	0.9506
	20	Y = 0.0071x + 0.3610	7.1 × 10^–3^	0.9310
	25	Y = 0.0188x + 0.1027	1.88 × 10^–2^	0.9888

### Effect of initial pH

**Figure 7:**
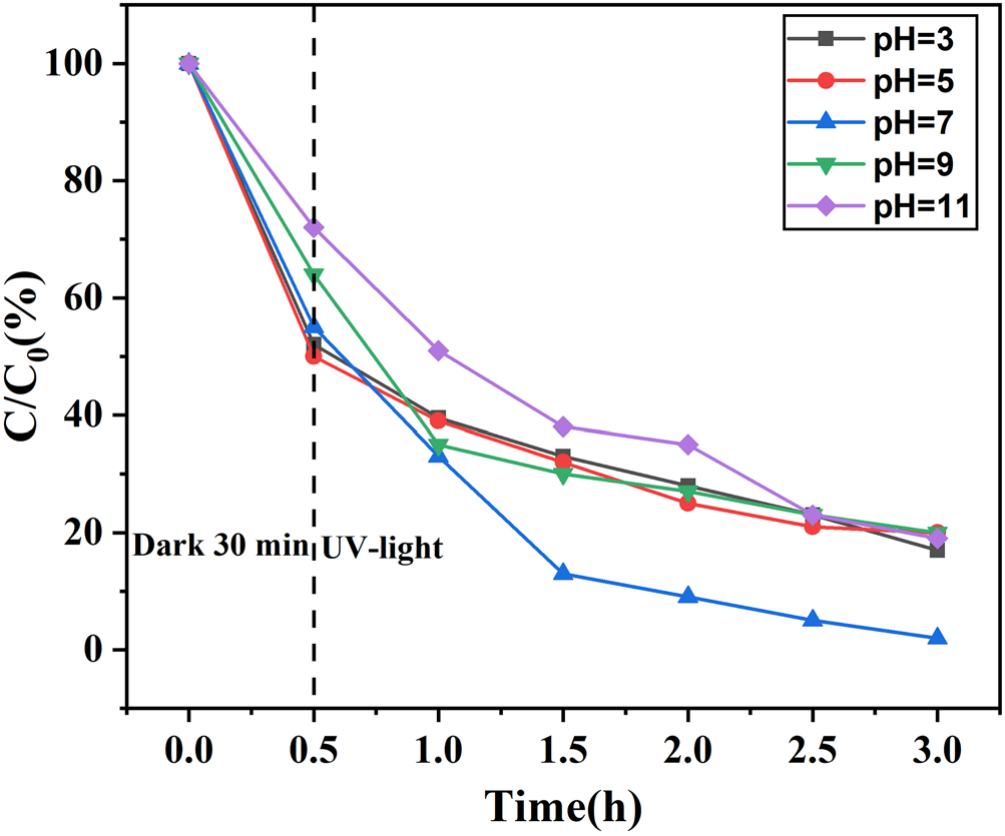
Effect of different pH on the degradation of NOF ([NOF] = 0.025 g L^−1^; [ZnO/ZnS@BC] = 0.5 g L^−1^; pH = 7).

The effect of pH on the removal of NOF by ZnO/ZnS@BC is shown in Fig. 7. When the sample was placed in the dark for 30 min, ZnO/ZnS@BC showed high adsorption capacity at pH 5 and low adsorption capacity at pH 11 with NOF removal rates of 50% and 28%, respectively. When under UV-light for 3 h at pH 7, the NOF removal rate reached a maximum of 96%. In alkaline conditions, especially at pH 11, the photocatalytic degradation rate was significantly reduced. The primary reason might be the different effects of pH on the adsorption of ZnO/ZnS@BC for the removal and photocatalytic degradation of NOF. On the one hand, the pK_a1_ and pK_a2_ of NOF were 6.20 and 8.70, respectively. NOF existed as a cation when pH < 6.20 and as an anion when pH > 8.70. When the solution was in a neutral state, NOF was in a neutral molecular state. Because of hydrophobic interaction, NOF was easily combined with the adsorption site on the surface of the adsorbent material^35^. On the other hand, the catalytic activity was closely related to the charged nature of the surface of the catalyst. The pH primarily affected the rate of photodegradation by changing the electrostatic interaction of the photocatalyst surface with solvent molecules, target degradation materials, and hydroxyl radicals^36^. The catalyst exhibited various eliminative capacities for NOF in different pH conditions. Other researchers found that pH is an important factor in the photocatalytic degradation of NOF, and removal rate reached an extreme value at pH 8.03 with visible photocatalytic degradation^37^. This was consistent with our results.

### Effect of different ZnO/ZnS loadings

**Figure 8:**
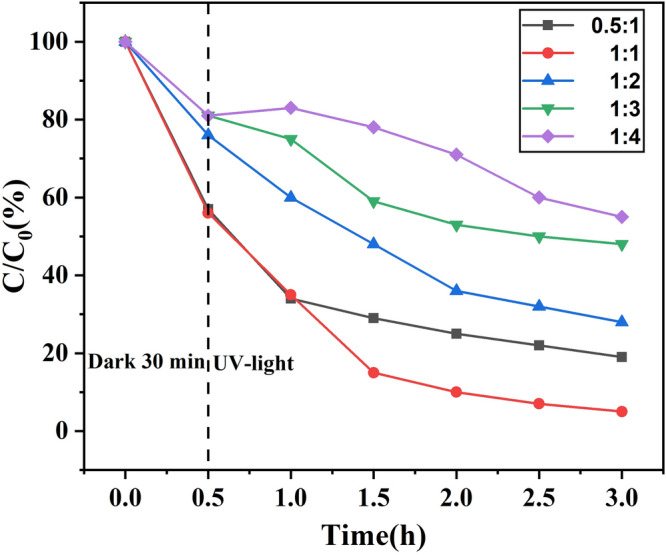
Effect of different ZnO/ZnS loadings on the catalytic degradation of NOR by ZnO/ZnS@BC under ultraviolet light conditions.

The ZnO/ZnS content with ratios of 0.5:1, 1:1, 2:1, 3:1, and 4:1 (ZnSO_4_:PS (m:m)) in the system were investigated to show the effect of the quantity of ZnO/ZnS on the catalytic activity of ZnO/ZnS@BC. The ZnO/ZnS@BC with a 1:1 ratio had the highest removal capacity in the dark and under UV-light, and the NOF adsorption and degradation rates were 44% and 49%, respectively (Fig. 8). This result reveals that the correct mole ratio of ZnO and ZnS in the compound built more efficient heterojunction nanostructures, and the heterojunctions significantly enhanced the photocatalytic performance, perhaps because the adsorption site and the photocatalytic active site reached an optimal ratio. With the increase of ZnO/ZnS, the removal rate of NOF gradually decreases. The ZnO/ZnS@BC with a 4:1 ratio had the lowest activity with 42% elimination after 3 h. This could be because of the decrease of adsorption sites on the surface of the ZnO/ZnS@BC. Thus, more catalytic active sites would be retained, enhancing the degradation of NOF.

### Effect of different amounts of ZnO/ZnS@BC

**Figure 9:**
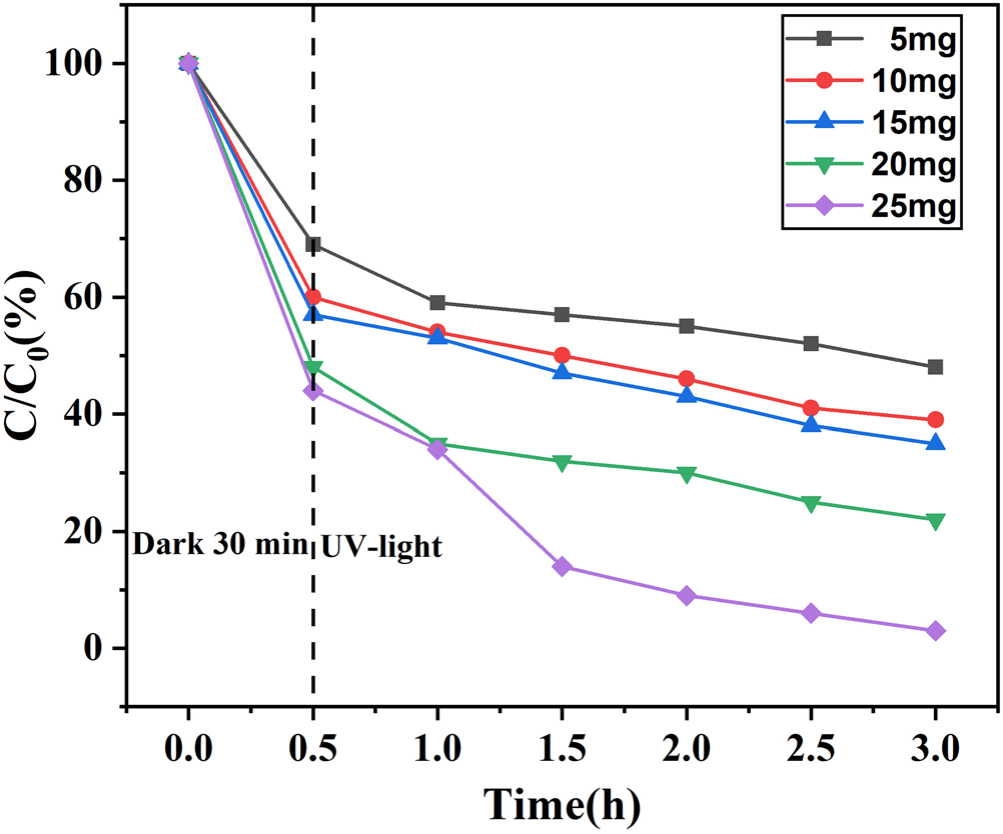
Effect of ZnO/ZnS@BC on the catalytic degradation of NOR by ZnO/ZnS@BC under ultraviolet light.

ZnO/ZnS@BC adsorbed and degraded NOF as adsorbent and photocatalytic material. Thus, the dosage was very important during the removal process. To investigate the effect of the amount of ZnO/ZnS@BC on the degradation, the effects of different dosages were explored (Fig. 9). When the dosages were 0.1 g L^−1^, 0.2 g L^−1^, 0.3 g L^−1^, 0.4 g L^−1^, and 0.5 g L^−1^, the adsorption rates of NOF in the dark were 30%, 40%, 42%, 52%, and 55%, respectively; under UV-light, the degradation rates of NOF were 18%, 16%, 18%, 28%, and 40%, respectively. When the dosages changed from 0.1 to 0.5 g L^−1^, the removal rate of NOF increased. This could be primarily attributed to adsorption and degradation. Because of the BC, ZnO/ZnS@BC possessed great adsorptive ability. Therefore, the removal efficiency of NOF should be raised by adding more catalysts, which could partly eliminate the influence of the extinction effect caused by the catalysts. Additionally, under the irradiation of the same ultraviolet light intensity, the generation of catalytic active substances increased as the amount of photocatalysts increased, enhancing the photocatalytic reaction. Tan’s studies indicated that both the adsorption and photodegradation of NOF were improved by increasing the photocatalyst amount^38^. A similar photocatalyst phenomenon was reported by other researchers; Chen found that the dosage of photocatalytic material had an important influence on the photocatalytic degradation rate because of the adsorption and catalysts.

### Effect of different competing ions

**Figure 10:**
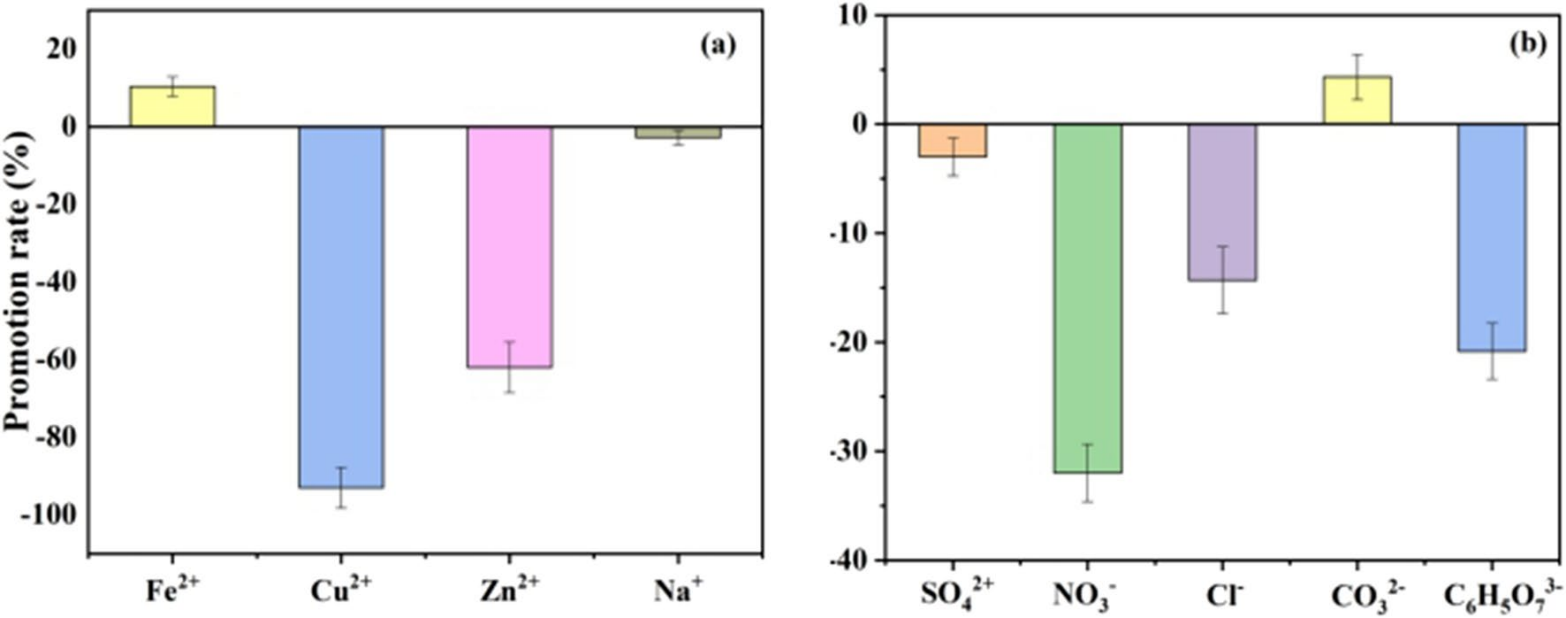
Effect of different ions on catalytic degradation of NOR by ZnO/ZnS@BC under ultraviolet light. (**a**) Effect of different cations on catalytic degradation. (**b**) Effect of different anions on catalytic degradation.

The effect of common cations (such as Na^+^, Fe^2+^, Cu^2+^, and Zn^2+^) and common anions (such as SO_4_^2−^, NO_3_^−^, Cl^−^, CO_3_^2−^, and C_6_H_5_O_7_^3−^) were investigated to study the effect of different ions on the removal of NOF by ZnO/ZnS@BC (results in Fig. 10). The concentration of the anions and cations were 0.1 mM, and the catalytic reaction time was 0.5 h. The influence of cations on the removal was significant (Fig. 10a). Fe^2+^ slightly promoted the removal effect (the promotion rate was 10% ), and Na^+^ had almost no impact (the inhibition rate was 2%). Other ions show an inhibitory effect. The inhibition rates of Cu^2+^ and Zn^2+^ were 99% and 62%, respectively. The primary reason was that the presence of cations forms a new clathrate with NOF, which was structurally stable and difficult to decompose. All the anions (except CO_3_^2−^, which slightly improved the removal) acted as inhibitors (Fig. 10b). CO_3_^2−^ ameliorated the pH of the solution, which improved the removal rate of NOF. SO_4_^2−^, NO^3−^, and Cl^−^ were formed associated with the consumption of the ·OH radical, which caused a decline in the degradation of the substances^37^. NO^3−^ had the highest inhibition rate of 34%. Additionally, anions might change the electrostatic interaction between NOF and ZnO/ZnS@BC. Therefore, the degradation rate of NOF was different in various ion solutions. The results were consistent with other research^39^.

### Reusability of the ZnO/ZnS@BC catalyst

**Figure 11:**
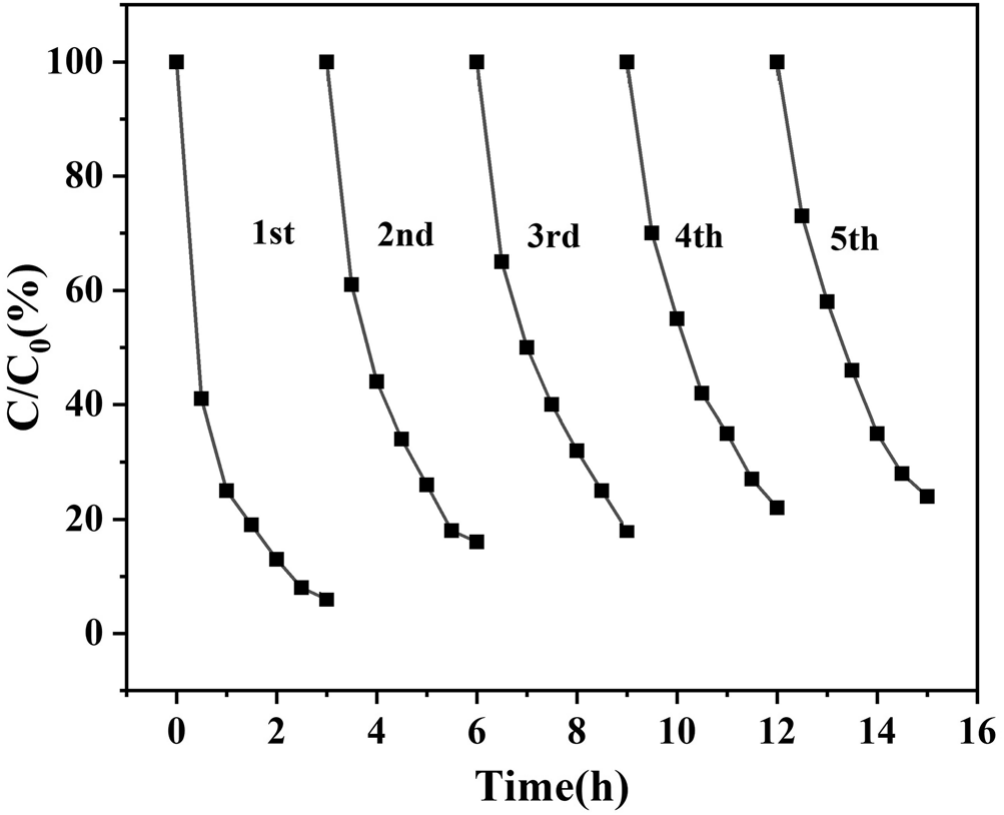
Repeat 5 times for ZnO/ZnS@BC recycling.

Stability was vital for the catalysts and was confirmed by repeating the decomposition processes five times (Fig. 11). After five replicates, the photodegradation efficiency of NOF decreased from 95 to 79%. The ZnO/ZnS@BC catalyst featured high stability and good reusability under ultraviolet light irradiation. The 16% reduction might be a result of the loss of catalyst quality during the recovery process.

### NOF degradation mechanisms

**Figure 12:**
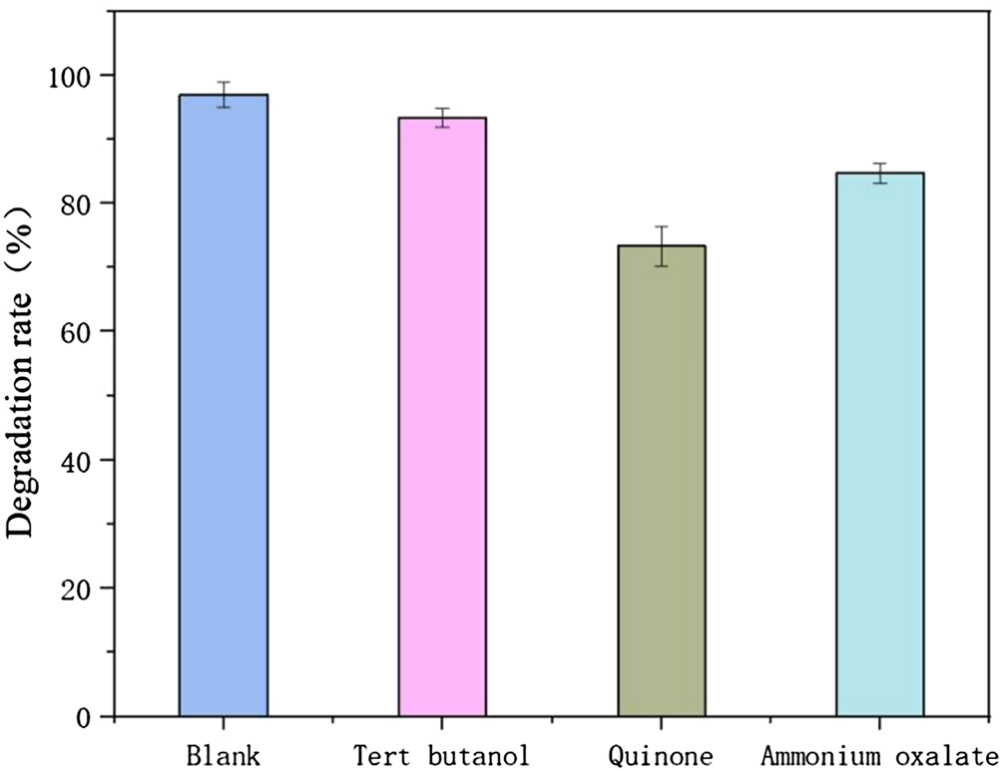
Effect of different quenchers on the catalytic degradation of NOR by ZnO/ZnS@BC under ultraviolet light.

Photogenerated h^+^, ·OH^−^, and superoxide (·O_2_^−^) were considered the primary active species responsible for the organic compound. In this research, tert-butanol, benzoquinone, and ammonium oxalate were used as scavengers of ·OH^−^, ·O_2_^−^, and h^+^, respectively^40^ (Fig. 12). Approximately 94% of the NOF was degraded within 3 h when there were no quenching agents in the system. However, there was an obvious decline of NOF photodegradation when benzoquinone was added—applied to quench ·O_2_^−^ in the system, and NOF removal declined by approximately 30%. Thus, the ·O_2_^−^ was generated in the photocatalytic reaction system when ZnO/ZnS@BC was irradiated by UV-light. This result also proved ·O_2_^−^ was the predominant active species in the system. In the presence of ammonium oxalate, which quenched h^+^, the removal of NOF was attenuated, and the removal rate of NOF was significantly reduced by 15%, which implied that h^+^ was also an important active species for NOF removal. Similarly, tert-butanol (used as a scavenger to quench) suppressed the photocatalytic removal rate by 7%, showing that ∙OH had a slight removal capacity.

Based on the results, each scavenger had a different effect on the photocatalytic degradation of NOF. Benzoquinone had the greatest influence, and tert-butanol had the weakest effect. The decreasing order of contribution of each reactive species on NOF degradation by ZnO/ZnS@BC was as follows: ·O_2_^−^ > ·OH^-^ > h^+^. This result was similar to research by Xian which used BaTiO3@g-C_3_N_4_ to degrade methyl orange under simulated sunlight irradiation^41^.

## Conclusion

In this study, the microscopic morphology and chemical substances of carbon-based composite photocatalysts were investigated by SEM, TEM, XPS and, EDS, respectively. The loading of abundant white granularity on the BC was directly observed by SEM imagery. According to the analysis of the XPS pattern, the white granularity was ZnO and ZnS. The effects of different experimental conditions on the photocatalytic degradation of NOF by ZnO/ZnS@BC were investigated. The results showed that the highest photocatalytic capability was achieved with a mass ratio of ZnSO_4_ and PS of 1:1 at pH 7; these conditions increased the degradation rate of NOF by 35.7%. The quenching experiment showed that ·OH, ·O_2_^−^, and h^+^ were the principal active substances in the degradation system and significantly contributed to the degradation of NOF; ·O_2_^−^ played the most important role. After recycling five times, the ZnO/ZnS@BC possessed good stability, and the degradation rate of NOF remained at 79%. Other studies also showed that carbon-based composites have a strong removal effect on NOF. A novel nanocomposite (N-RGO/Fe_3_O_4_) was synthesised by a facile hydrolysis process and possessed the function of adsorption and catalytic degradation of NOF in water. Peng synthesised a composite of Fe/Fe_3_C and N-doped graphitic carbon (Fe/Fe_3_C@NG) and used it as a catalyst to degrade NOF. The results showed that the composite possessed good adsorption capacity and catalytic activity and had a high removal rate for norfloxacin. Three primary factors influenced NOF removal. Firstly, BC quickly adsorbed the NOF on its surface and increased the contact probability of the NOF and the catalyst. Secondly, during the process of calcination, BC increased the mechanical strength of the catalysts to protect ZnO/ZnS from aggregation. Therefore, ZnO/ZnS was supported on the surface of the BC, and the active area was enlarged, which enhanced catalytic activity. A reasonable ratio of adsorptive sites to catalytic sites, which leads to strong synergistic effects of adsorptive and catalysis, is crucial. Thirdly, ZnO and ZnS formed heterojunctions, increasing the dispersion of the hole-electron pairs. This was the principal method to enhance catalytic performance. Thus, the catalysts highly accelerated the reaction rate to reduce the production of by-products which were probably toxic. Therefore, ZnO/ZnS@BC is a good material for the catalytic degradation of NOF in water and has favourable application prospects in sewage treatment systems.
